# High Genetic Diversity and Adaptive Potential of Two Simian Hemorrhagic Fever Viruses in a Wild Primate Population

**DOI:** 10.1371/journal.pone.0090714

**Published:** 2014-03-20

**Authors:** Adam L. Bailey, Michael Lauck, Andrea Weiler, Samuel D. Sibley, Jorge M. Dinis, Zachary Bergman, Chase W. Nelson, Michael Correll, Michael Gleicher, David Hyeroba, Alex Tumukunde, Geoffrey Weny, Colin Chapman, Jens H. Kuhn, Austin L. Hughes, Thomas C. Friedrich, Tony L. Goldberg, David H. O'Connor

**Affiliations:** 1 Department of Pathology and Laboratory Medicine, University of Wisconsin–Madison, Madison, Wisconsin, United States of America; 2 Wisconsin National Primate Research Center, Madison, Wisconsin, United States of America; 3 Department of Pathobiological Sciences, University of Wisconsin–Madison, Madison, Wisconsin, United States of America; 4 Department of Biological Sciences, University of South Carolina, Columbia, South Carolina, United States of America; 5 Department of Computer Sciences, University of Wisconsin–Madison, Madison, Wisconsin, United States of America; 6 Makerere University, Kampala, Uganda; 7 Department of Anthropology and School of Environment, McGill University, Montreal, Quebec, Canada; 8 Integrated Research Facility at Fort Detrick, National Institute of Allergy and Infectious Diseases, National Institutes of Health, Fort Detrick, Frederick, Maryland, United States of America; German Primate Center, Germany

## Abstract

Key biological properties such as high genetic diversity and high evolutionary rate enhance the potential of certain RNA viruses to adapt and emerge. Identifying viruses with these properties in their natural hosts could dramatically improve disease forecasting and surveillance. Recently, we discovered two novel members of the viral family *Arteriviridae*: simian hemorrhagic fever virus (SHFV)-krc1 and SHFV-krc2, infecting a single wild red colobus (*Procolobus rufomitratus tephrosceles*) in Kibale National Park, Uganda. Nearly nothing is known about the biological properties of SHFVs in nature, although the SHFV type strain, SHFV-LVR, has caused devastating outbreaks of viral hemorrhagic fever in captive macaques. Here we detected SHFV-krc1 and SHFV-krc2 in 40% and 47% of 60 wild red colobus tested, respectively. We found viral loads in excess of 10^6^–10^7^ RNA copies per milliliter of blood plasma for each of these viruses. SHFV-krc1 and SHFV-krc2 also showed high genetic diversity at both the inter- and intra-host levels. Analyses of synonymous and non-synonymous nucleotide diversity across viral genomes revealed patterns suggestive of positive selection in SHFV open reading frames (ORF) 5 (SHFV-krc2 only) and 7 (SHFV-krc1 and SHFV-krc2). Thus, these viruses share several important properties with some of the most rapidly evolving, emergent RNA viruses.

## Introduction

Certain RNA viruses have biological properties that make them particularly likely to emerge [1]. High genetic diversity, high evolutionary rates, and high viral loads are all thought to enhance the potential of some RNA viruses to adapt to changing environments by evading immune responses within hosts or enabling the invasion of new host populations [2], [3]. It is widely accepted that identifying and characterizing such viruses in their natural hosts is important for disease monitoring and prevention [4]–[7]. For example, the origin of human immunodeficiency virus (HIV)-1, group M (the strain responsible for the AIDS pandemic) from simian immunodeficiency viruses (SIVs) of wild chimpanzees in Central Africa [8] underscores the importance of “pandemic prevention,” as well as the importance of non-human primates as reservoirs of potentially important viruses.

The simian hemorrhagic fever viruses (SHFVs) are a poorly understood group of single stranded, positive-sense RNA viruses within the family *Arteriviridae* that have only recently been detected in wild primates [9], [10]. Almost everything known about these viruses comes from the type strain of simian hemorrhagic fever virus (SHFV-LVR), which caused several “explosive” disease outbreaks in captive macaques (*Macaca assamensis, M. arctoides, M. fasciularis, M. nemestrina, and M. mulatta*) between 1964 and 1996. [11]–[14]. The lethality of SHFV infection in these Asian Old World monkeys (OWMs) suggested that macaques were highly susceptible to the virus, and were therefore unlikely to be natural hosts of SHFV-LVR. Further investigation revealed that monkeys of several African OWM species – specifically patas monkeys (*Erythrocebus patas*), grivets (*Chlorocebus aethiops*), and Guinea baboons (*Papio papio*) – could persistently harbor SHFV-LVR in captivity without signs of disease [15]. Although this finding implicated African OWMs as the immediate source of SHFV-LVR in the captive outbreaks, neither SHFV-LVR nor any of its relatives had ever been identified in a wild animal until recently [9], [10], [16].

In 2011, we discovered two highly divergent simian arteriviruses infecting a single wild red colobus (*Procolobus rufomitratus tephrosceles*) in Kibale National Park, Uganda (hereafter Kibale), which we named SHFV-krc1 and SHFV-krc2 [9]. Subsequently, we discovered additional, highly divergent simian arteriviruses in red-tailed guenons (*Cercopithecus ascanius*) from the same location [10]. Here we characterize SHFV-krc1 and SHFV-krc2 in 60 red colobus from Kibale. We show that these viruses infect a high proportion of red colobus in this population, replicate to high titers in infected monkeys, and have high genetic diversity, both within and among hosts. Our findings demonstrate that these viruses possess properties that are associated with the rapid evolutionary adaptability characteristic of many emerging RNA viruses.

## Materials and Methods

### Arterivirus genome organization and nomenclature

**Figure 1:**
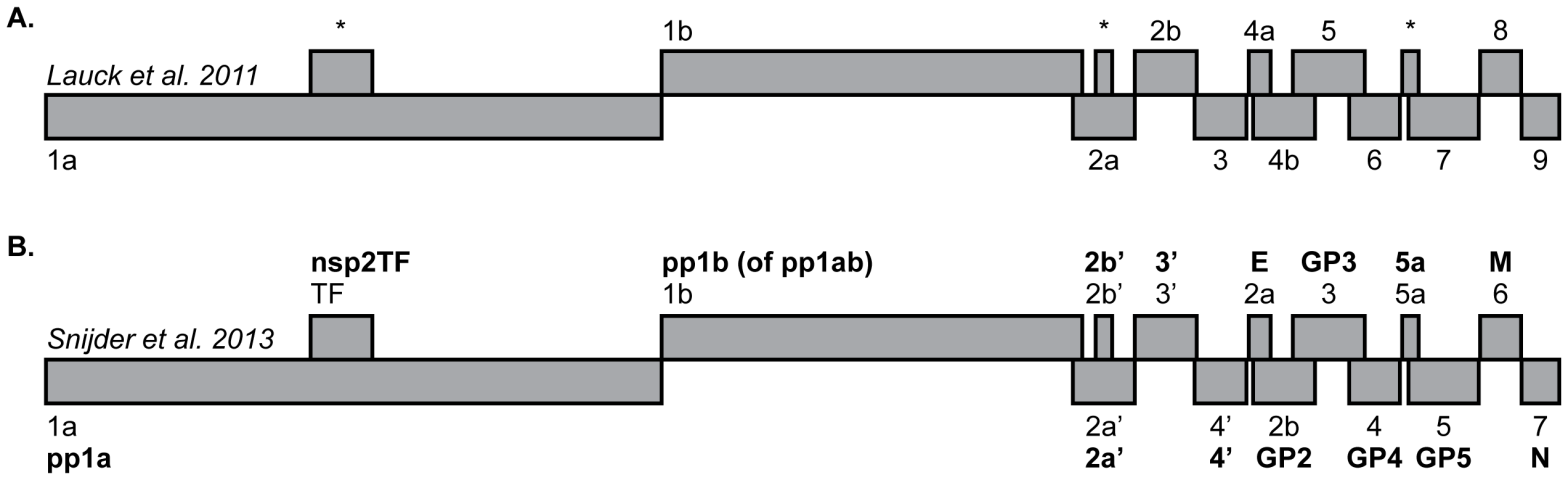
Schematic of the SHFV genome. (A) ORFs as they are referred to in Lauck et al., 2011 [9], labeled sequentially 5′-3′: ORF1a-ORF9. Asterisks denote ORFs identified in SHFV-krc1 and SHFV-krc2 not reported in Lauck et al., 2011 [9]. (B) ORFs as they are named in Snijder et al., 2013 [17], labeled 5′-3′: ORF1a-ORF7, with duplicated ORFs designated by a “prime” (*e.g.* ORF2a’). Expression products are given in bold.

SHFV genomes contain a duplication of four open reading frames (ORFs) relative to the other viruses in the *Arteriviridae* family: porcine reproductive and respiratory syndrome virus (PRRSV), equine arteritis virus (EAV), and lactate dehydrogenase-elevating virus of mice (LDV). Previous publications regarding SHFV have treated the naming of these additional ORFs inconsistently. For clarity, we have adopted the nomenclature scheme presented in [17], and have included a schematic (Figure 1) to maintain continuity with previous publications.

### Ethics statement

All animal use in this study followed the guidelines of the Weatherall Report on the use of non-human primates in research. Specific protocols were adopted to minimize suffering through anesthesia and other means during capture, immobilization, and sampling of the non-human primates. These included use of anesthesia during capture (Ketamine/Xylazine, administered intramuscularly with a variable-pressure pneumatic rifle), minimization of immobilization time and the use of an anesthetic reversal agent (Atipamezole) to reduce recovery time, and conservative limits on blood sample volumes (<1% body weight), as previously described [9]. Following sampling, all animals were immediately released back to their social group without incident [18]. All research was conducted on public land and approved by the Uganda Wildlife Authority (permit UWA/TDO/33/02), the Uganda National Council for Science and Technology (permit HS 364), and the University of Wisconsin Animal Care and Use Committee (protocol V01409-0-02-09) prior to initiation of the study.

### Study site and sample collection

Red colobus were sampled between 2/5/2010 and 7/22/2012 in Kibale National Park, Uganda, a 795 km^2^ semi-deciduous park in western Uganda (0°13′–0°41′N, 30°19′–30°32′E) known for its exceptional density of primates belonging to diverse species. Blood was separated using centrifugation and plasma was frozen immediately in liquid nitrogen for storage and transport to the United States. Samples were shipped in an IATA-approved dry shipper to the USA for further analysis at the Wisconsin National Primate Research Center in accordance with CITES permit #002290 (Uganda).

### Molecular methods

Samples were processed for sequencing in a biosafety level 3 laboratory as described previously [9], [10]. Briefly, for each animal, one ml of blood plasma was filtered (0.45 µm) and viral RNA was isolated using the Qiagen QIAamp MinElute virus spin kit (Qiagen, Hilden, Germany), omitting carrier RNA. DNase treatment was performed and cDNA synthesis was accomplished using random hexamers. Samples were fragmented and sequencing adaptors were added using the Nextera DNA Sample Preparation Kit (Illumina, San Diego, CA, USA). Deep sequencing was performed on the Illumina MiSeq (Illumina, San Diego, CA, USA).

### SHFV detection by quantitative RT-PCR

We developed a multiplex quantitative RT-PCR (qRT-PCR) assay to quantify plasma viral RNA of both SHFV-krc1 and SHFV-krc2 in each sample. Taqman assays were designed with amplification primers specific for either SHFV-krc1 (5′-ACACGGCTACCCTTACTCC-3′ and 5′- TCGAGGTTAARCGGTTGAGA-3′) or SHFV-krc2 (5′-AACGCGCACCAACCACTATG-3′ and 5′GCGTGTTGAGGCCCTAATTTG-3′). The SHFV-krc1 probe (5′-Quasar 670-TTCTGGTCCTCTTGCGAAGGC-BHQ2-3′) and SHFV-krc2 probe (5′-6-Fam-TTTGCTCAAGCCAATGACCTGCG-BHQ1-3′) were also virus-specific. The fluorophores used do not produce overlapping spectra, so no color compensation was required. Viral RNA was reverse transcribed and quantified using the SuperScript III One-Step qRT-PCR system (Invitrogen, Carlsbad, CA) on a LightCycler 480 (Roche, Indianapolis, IN). Reverse transcription was carried out at 37°C for 15 min and then 50°C for 30 min followed by two minutes at 95°C and 50 cycles of amplification as follows: 95°C for 15 sec and 60°C for 1 min. The reaction mixture contained MgSO^4^ at a final concentration of 3.0 mM, 150 ng random primers (Promega, Madison, WI), with all 4 amplification primers at a concentration of 600 nM and both probes at a concentration of 100 nM.

### Genetic analyses

Sequence data were analyzed using CLC Genomics Workbench 5.5 (CLC bio, Aarhus, Denmark) and Geneious R5 (Biomatters, Auckland, New Zealand). Low quality (<Q25) and short reads (<100 bp) were removed and the full genome sequences for each virus were acquired using *de novo* assembly. Due to the approximately 52% nucleotide sequence similarity between the genomes of SHFVkrc1 and SHFV-krc2, and the high frequency of co-infections in our animal cohort, we devised a method to minimize mapping of SHFV-krc1 reads to SHFV-krc2 (and vice versa) within a co-infected animal. Briefly, total reads from a co-infected animal were mapped to the SHFV-krc1 consensus sequence generated from *de novo* assembly and “unmapped reads” were collected, then mapped to the SHFV-krc2 consensus sequence obtained from *de novo* assembly. The resulting SHFV-krc2 consensus sequence was then used as the reference for mapping and collecting unmapped reads to map to the SHFV-krc1 consensus sequence generated from *de novo* assembly. This process was repeated until changes between the reference and the consensus sequences were not observed for either virus. Using this method, reads corresponding to SHFV-krc1 and SHFV-krc2 were reliably segregated in co-infected animals, with less than 0.2% of SHFV-specific reads mapping to both viruses. The average coverage per genome was 5,654× (range 118-19,115×) for SHFV-krc1 variants and 2,264 (range 94-6,613×) for SHFV-krc2 variants. For intra-host genetic analysis, sequencing reads were mapped to the corresponding consensus sequence for each variant. Single nucleotide polymorphism (SNP) reports were generated in Geneious, with a minimum coverage threshold of 100 reads and a minimum frequency threshold of five percent.

### Evolutionary analyses

The synonymous nucleotide diversity (π_S_) and the non-synonymous nucleotide diversity (π_N_) were estimated for each ORF individually from SNP reports generated by mapping sequencing reads to their corresponding consensus sequence. We estimated π_S_ = n_s_/L_s_ and π_N_ = n_n_/L_n_, where n_s_ is the mean number of pairwise synonymous differences; n_n_ is the mean number of pairwise synonymous differences; L_s_ is the number of synonymous sites; and L_n_ is the number of nonsynymous sites. L_s_ and L_n_ were estimated by the method described in [19]. To compare viruses across different hosts, variant consensus sequences were aligned by the CLUSTAL algorithm in MEGA 5.05 [20]. Estimating π_S_ and π_N_ separately for each ORF in each virus from co-infected animals, we used a factorial analysis of variance to test for main effects of the virus (SHFV-krc1 vs. SHFV-krc2) and the ORF, and for virus-by-ORF interactions. In the case of π_S_, there were highly significant main effects of virus (F_1, 459_ = 41.31; *p*<0.001) and of ORF (F_13, 459_ = 14.07; *p*<0.001), but there was not a significant virus-by-ORF interaction (F_13, 459_ = 1.35; n.s.). In the case of π_N_, there were significant main effects of virus (F_1, 459_ = 4.42; *p* = 0.036) and of ORF (F_13, 459_ = 53.26; *p*<0.001), and there was a highly significant virus-by-ORF interaction (F_13, 459_ = 4.39; *p*<0.001). Sliding window analysis involved estimating π_S_ and π_N_ in a sliding window of 9 codons, numbered according to the numbering in the sequence alignment of the first codon in the window.

### Layercake visualization

We developed a specialized visualization tool called LayerCake for this dataset. This tool allows visual comparison of variants for multiple individuals simultaneously, encoding sequences as bands of color, with redder sections of the band corresponding to regions with a higher proportion of polymorphic reads. Downloadable versions of the krc1 and krc2 datasets are available, along with a generalized tutorial for interpreting LayerCake displays, at http://graphics.cs.wisc.edu/Vis/LayerCake/.

## Results

### Sample collection and infection frequency of SHFV-krc1 and SHFV-krc2 in Kibale red colobus

**Figure 2:**
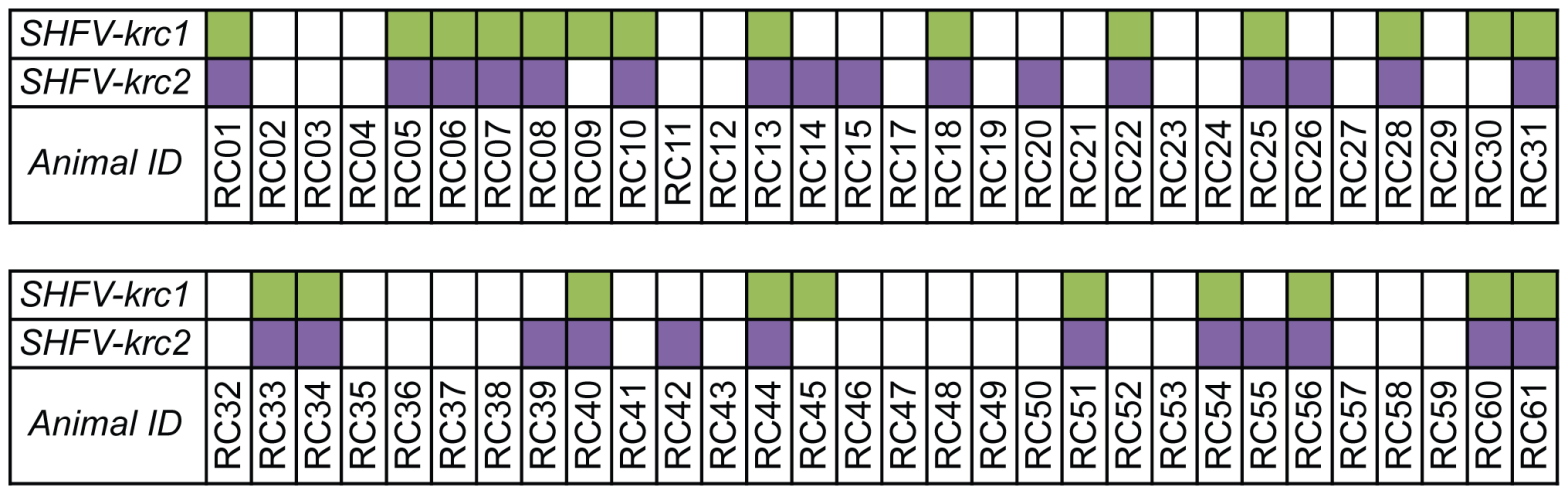
Infection frequency of SHFV-krc1 and SHFV-krc2 in the Kibale red colobus. SHFV-krc1 (green) and SHFV-krc2 (purple) infections were identified by “unbiased” deep sequencing and confirmed by strain-specific qRT-PCR.

Blood samples were collected from 60 adult red colobus residing in the Kanyawara area of Kibale over a period of 2.5 years. These animals represent approximately half of a defined social group, but comprise a relatively small proportion of the total red colobus population in Kibale [21]. All animals appeared normal and healthy at the time of sampling. RNA was isolated from the blood plasma of each animal and “unbiased” deep sequencing was performed on an Illumina MiSeq machine as previously described [9], [10]. *De novo* assembly and iterative mapping of sequencing reads yielded 52 near full-length SHFV consensus sequences (GenBank accession numbers KC787607-KC787658). Twenty-four animals (40.0%) were infected with SHFV-krc1, and 28 animals (46.7%) were infected with SHFV-krc2. Twenty-one animals (35.0%) were co-infected with both SHFV-krc1 and SHFV-krc2 (Figure 2).

### Viral loads of SHFV-krc1 and SHFV-krc2 in the Kibale red colobus

**Figure 3:**
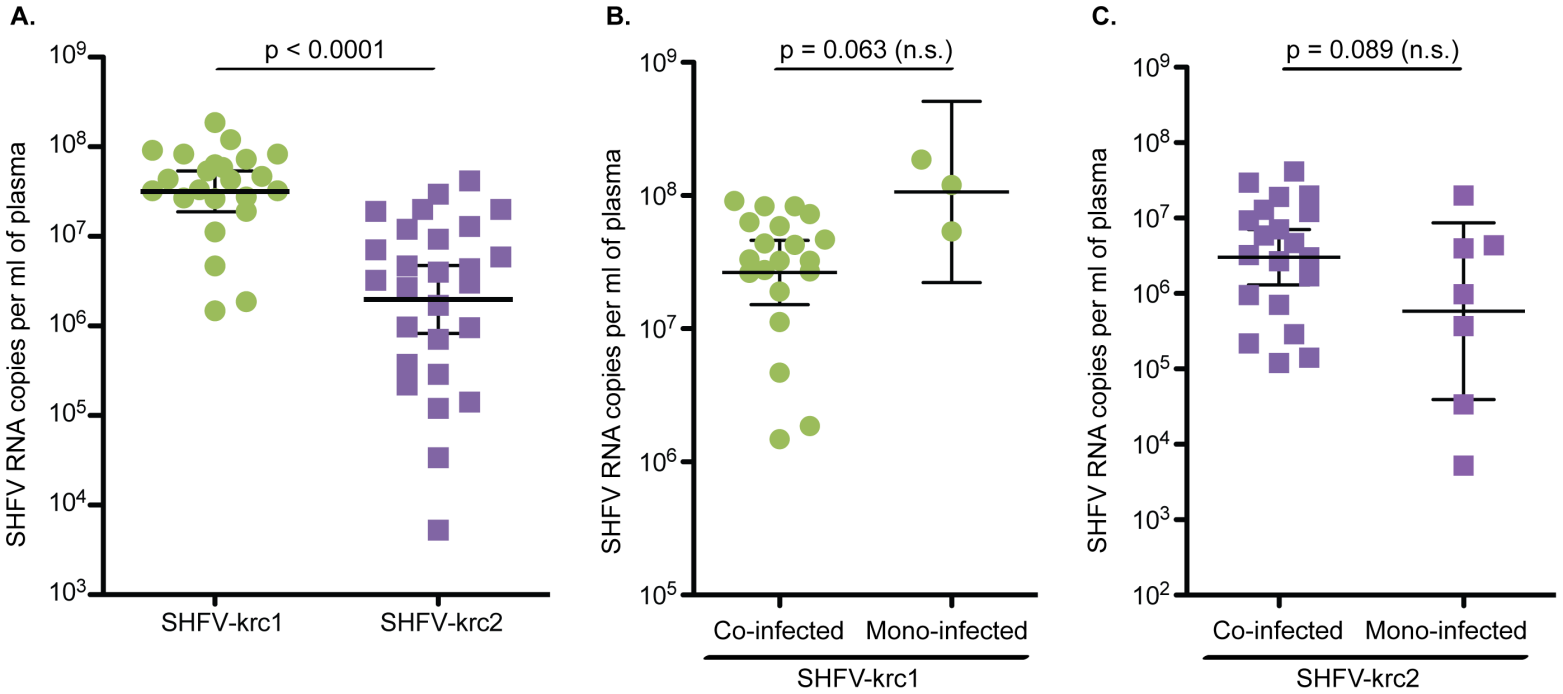
Viral loads of SHFV-krc1 and SHFV-krc2 in the Kibale red colobus. Comparison of SHFV-krc1 (green) and SHFV-krc2 (purple) viral loads from all animals positive for either virus (A) and viral loads from mono-infections vs. co-infections of SHFV-krc1 (B) and SHFV-krc2 (C). RNA was isolated from blood plasma and quantitative RT-PCR was performed using strain-specific primers and probes designed from deep sequencing data. Statistical significance was assessed using a two-tailed t-test performed on log-transformed values (CI = 95%).

To estimate the viral load of SHFV-krc1 and SHFV-krc2 in infected red colobus, a strain-specific qRT-PCR assay was designed to amplify highly conserved regions in ORF7 of the SHFV-krc1 and SHFV-krc2 genomes. This assay was used to assess the viral burden in cell-free plasma for each animal found to be positive by deep sequencing. SHFV-krc1 viremia was consistently high, averaging 5.1×10^7^ vRNA copies/ml plasma, (range: 1.5×10^6^–1.9×10^8^ copies/ml plasma) (Figure 3A). SHFV-krc2 loads were more varied (range: 3.4×10^4^–4.1×10^7^ copies/ml) and significantly lower than SHFV-krc1 with an average plasma titer of 7.5×10^6^ vRNA copies/ml plasma (*p* = 0.0001, two-tailed t-test). Although instances of mono-infection were scarce relative to co-infection, mono/co-infection status did not impact the load of either virus to a statistically significant extent (mono- vs. co-infected: *p* = 0.063 for SHFV-krc1, *p* = 0.089 for SHFV-krc2, two-tailed t-test, Figure 3B,C).

### Consensus-level genetic diversity among SHFV-krc1 and SHFV-krc2 variants

**Figure 4:**
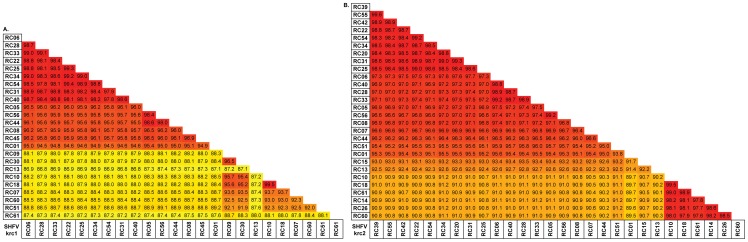
Pairwise comparison of nucleotide identity among variants of SHFV-krc1 and SHFV-krc2 from Kibale red colobus (RC). Full coding sequences for each isolate were aligned using CLC Genomics Workbench. Numbers show percent nucleotide identity between two variants within (A) SHFV-krc1 or (B) SHFV-krc2. Colors highlight similarity, with red representing the most similar sequences and yellow representing sequences with the lowest degree of nucleotide identity. The same color scale was used for (A) and (B).

To quantify the genetic diversity of SHFV-krc1 and SHFV-krc2 within the red colobus population, we examined similarity among the 24 SHFV-krc1 and 28 SHFV-krc2 variants by comparing the nucleotide consensus sequences of each viral variant (Figure 4). These consensus sequences represent the majority nucleotide base present at each position of the genome for the viral population within each host. Because RNA viruses often exist within a host as a highly heterogeneous population (*i.e.* “mutant swarm”), the consensus sequence may not actually be present in the within-host viral population [22], [23]. Nevertheless, the construction of consensus sequences allowed us to compare the average viral population of each variant (*i.e.* inter-host diversity). For SHFV-krc1, percent pairwise nucleotide identity between variants ranged from 86.9%–99.5%. A highly related core group (SHFV-krc1 from red colobus 06, 28, 33, 22, 25, 34, 54, 31, 40, 05, 56, 44, 08, 45, 01) with pairwise nucleotide identities ranging from 94.5%–99.3% comprised 63% of the variants (Figure 4A). A distinct second group (SHFV-krc1 from red colobus 09, 30, 10, 18, 07, 60) with a slightly wider range of similarity (92.0%–99.5% pairwise nucleotide identity) made up an additional 21% of variants. A similar pattern, with two distinct groups, was observed for SHFV-krc2 variants (range: 89.67%–99.48% pairwise nucleotide identity, Figure 4B). However, patterns of SHFV-krc1 genetic similarity among hosts were different from patterns of SHFV-krc2 similarity among hosts. (Figure 4A,B). Interestingly, SHFV-krc1 variants from red colobus 13 and 61 were highly dissimilar to all other SHFV-krc1 variants identified, with pairwise nucleotide identities ranging from 86.8%–88.7%.

### Within-host genetic diversity of SHFV-krc1 and SHFV-krc2

**Figure 5:**
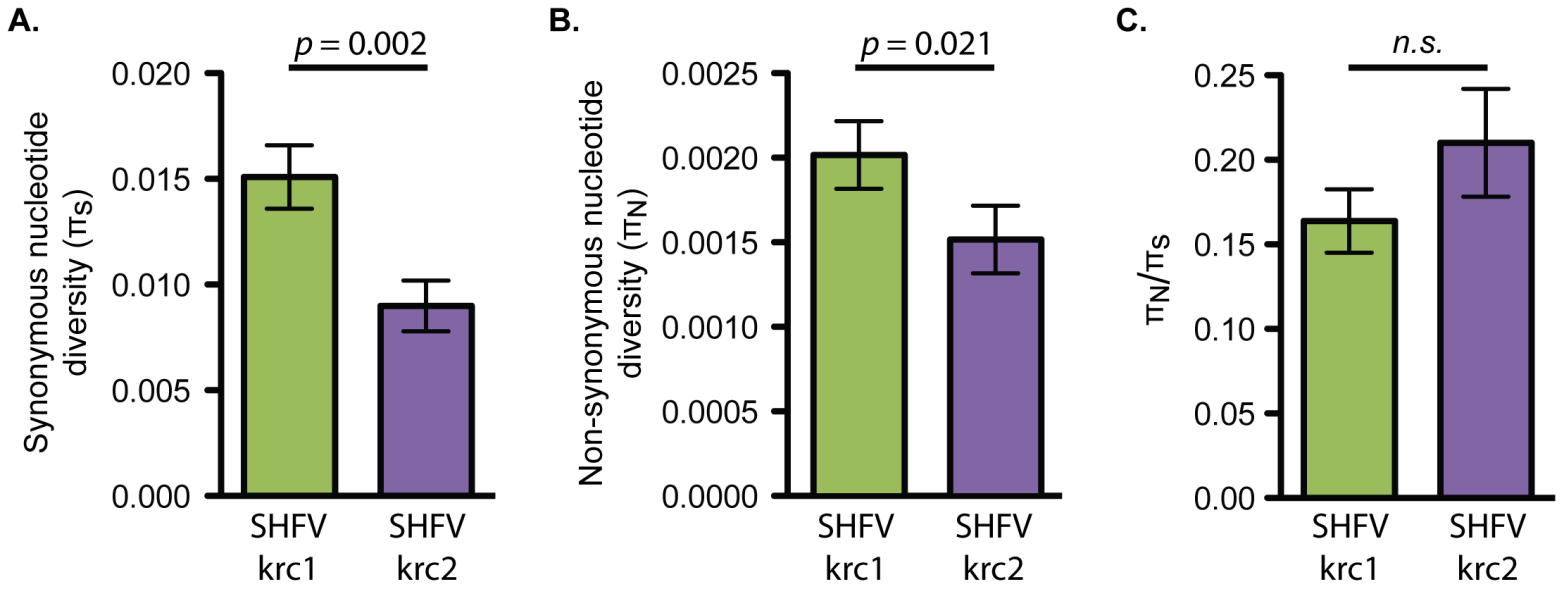
Overall nucleotide diversity of SHFV-krc1 and SHFV-krc2. Mean (± S.E.) π_S_ (A), π_N_ (B), and π_N_/π_S_ (C) in monkeys infected with SHFV-krc1 (green) and SHFV-krc2 (purple). Paired t-tests were performed to compare mean values between SHFV-krc1 and SHFV-krc2.

To examine the genetic diversity of SHFV-krc1 and SHFV-krc2 within individual monkeys, we calculated the non-synonymous and synonymous nucleotide diversity, π_N_ and π_S_ respectively, for each within-host viral population using deep sequencing reads from each viral variant. Comparing π_N_ and π_S_ from specific regions of a viral genome can reveal the mode of natural selection acting on a region. For example, π_N_<π_S_ is indicative of negative selection acting to remove deleterious protein-coding mutations, while π_N_>π_S_ is suggestive of positive selection acting to drive beneficial protein-coding mutations to fixation. We found that, overall, negative selection acting against deleterious non-synonymous mutations predominated for both SHFV-krc1 and SHFV-krc2. In SHFV-krc1, π_S_ exceeded π_N_ by a ratio of over 6∶1, whereas in SHFV-krc2, π_S_ exceeded π_N_ by a ratio of nearly 5∶1. Both π_S_ and π_N_ were significantly greater in SHFV-krc1 than in SHFV-krc2 (*p* = 0.002 and *p* = 0.021, paired t-test), indicating greater overall nucleotide diversity in SHFV-krc1 than in SHFV-krc2 (Figure 5). A positive correlation between viral load and both π_S_ and π_N_ was observed. However, mean π_S_ and π_N_ did not differ significantly between co-infected monkeys and those infected with only SHFV-krc1 or SHFV-krc2 (data not shown).

**Figure 6:**
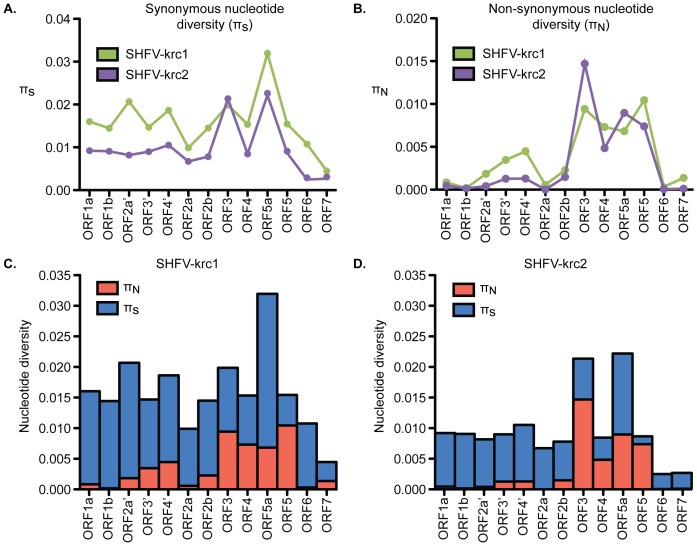
Nucleotide diversity of SHFV-krc1 and SHFV-krc2 by ORF. Interaction graphs comparing mean π_S_ (A) and π_N_ (B) in ORFs from SHFV-krc1 (green) and SHFV-krc2 (purple). In the case of πN there was a significant ORF-by-virus interaction (F13, 459 = 4.39; p<0.001). Comparison of mean πs (blue) to πN (red) within ORFs of SHFV-krc1 (C) and SHFV-krc2 (D) revealed substantial differences among ORFs within each virus.

**Figure 7:**
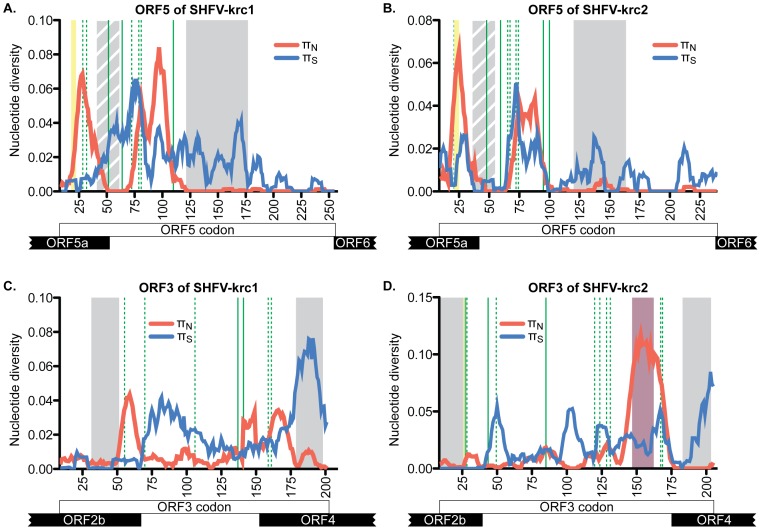
Nucleotide diversity across ORF3 and ORF5 of SHFV-krc1 and SHFV-krc2. Mean π_S_ (blue) and π_N_ (red) in sliding windows of 9 codons across the coding region of ORF5 (A,B) and ORF3 (C,D). Overlapping ORFs are shown at the bottom. Grey boxes represent predicted transmembrane domains, with striped grey boxes representing a hydrophobic region unique to the SHFVs. Green lines depict putative sites of *N*-glycosylation, with dashed green lines showing sites that are variably glycosylated. Yellow boxes show predicted signal peptide cleavage sites that vary in location in GP5 of SHFV-krc1 and SHFV-krc2 and were not found in GP3 of SHFV-krc1. The purple box corresponds to the unique region of highly variable acidic residues found only in ORF3 of SHFV-krc2.

The organization of ORFs in the genomes of SHFV-krc1 and SHFV-krc2 was the same as described previously (Figure 1) [9], [10], [17], so we used a factorial analysis of variance approach to investigate π_S_ and π_N_ in ORFs in both viruses. In general, 3′-proximal ORFs displayed more non-synonymous diversity than 5′-proximal ORFs, suggesting that the proteins encoded by 5′-proximal ORFs may be more functionally constrained than those encoded by 3′-proximal ORFs. However, the extent to which underlying RNA structures may have affected this analysis is unknown [24]–[26]. ORF5 showed the highest mean π_N_ in SHFV-krc1 and among the highest in SHFV-krc2 (Figure 6). In the case of both SHFV-krc1 and SHFV-krc2, a sliding window plot of 9 codons revealed peaks of π_N_ corresponding to codons 1–46 and 64–100 of ORF5 (Figure 7A,B). The latter peak (codons 64–100) also involved high π_S_, suggesting a mutational hotspot. Interestingly, π_N_ was substantially higher in ORF3 of SHFV-krc2 than of SHFV-krc1 (Figure 6). Sliding window analysis revealed a substantial peak of π_N_ between codons 141–173 of SHFV-krc2 ORF3 (Figure 7C) that greatly exceeded π_S_, suggesting strong positive selection in this region of SHFV-krc2. This peak of π_N_ corresponded to a region of variable length rich in acidic residues. An analogous peak of π_N_ in ORF3 of SHFV-krc1 was not found, although a unique peak of π_N_ was identified between codons 50 and 68 (Figure 7D). Of note, a high degree of variability in predicted *N*-glycosylation [27] was associated with each instance of elevated π_N_ in ORF3 and ORF5 for both SHFV-krc1 and SHFV-krc2. For peaks of π_N_ found in regions of ORF3 and ORF5 that shared sequence with an overlapping alternative ORF, sliding window plot analysis in the alternative ORFs revealed peaks of π_S_ demonstrating that observed elevations in π_N_ were ORF-specific, as expected [28], [29] (data not shown).

Unique patterns of inter- and intra-host variation can be visualized on a genome-wide scale for all SHFV-krc1 and SHFV-krc2 variants using our custom-built LayerCake software: http://graphics.cs.wisc.edu/Vis/LayerCake/.

## Discussion

This study provides the first systematic analysis of SHFV genetic diversity in a population of wild non-human primates. Our findings show that SHFV-krc1 and SHFV-krc2 have a high frequency of infection in the red colobus population of Kibale, and that these viruses achieve high titers in the blood of infected monkeys. Our study also details, for the first time, the genetic diversity of SHFV-krc1 and SHFV-krc2 both within and among infected hosts. We draw particular attention to the signatures of natural selection identified throughout the genomes of these viruses, with an emphasis on signatures of positive selection identified in ORFs 3 and 5.

To date, primates from only two species – the red colobus and red-tailed guenon – have been found to harbor simian arteriviruses in the wild [9], [10]. Although these two species frequently associate with each other in the wild, including coming into direct contact [30], there is no evidence of SHFV transmission between red colobus and sypmatric red-tailed guenons. Despite this, the origins and host-ranges of these viruses are far from clear. Our findings support the hypothesis that simian arteriviruses are endemic to African OWMs and cause little to no clinical disease in these hosts. However, when introduced into Asian OWMs, these viruses may be lethal, as exemplified by SHFV-LVR [13], [31]. This pattern of pathogenesis is similar to SIV [32] and, like SIV, the simian arteriviruses appear to be well host-adapted, which suggests an ancient evolutionary relationship between these viruses and their African OWM hosts. This is in contrast to the arterivirus PRRSV, which emerged suddenly in pig populations across the globe in the 1980's [33]. Taken together, this implies that the prevalence and diversity of the *Arteriviridae*, including the simian arterivirus group, may be greater than currently appreciated.

SHFV-krc1 and SHFV-krc2 display many biological properties associated with the potential for rapid evolution – a feature that is shared by many emergent RNA viruses. For example, high diversity at the population level (inter-host diversity) can facilitate speciation, and related yet distinct viruses can recombine [32], [34]. High within-host diversity also enables a virus to escape the host immune response, alter tropism, and infect new host species [35], [36]. In these contexts, high viral load increases the probability of transmission by “widening” the population bottleneck that often reduces the fitness of an RNA virus upon transmission [37]–[39]. Such features enhance the ability of a virus to adapt to changing environments and have been implicated in the ability of some viruses to transmit across species barriers [2]. Although the arteriviruses in general are considered to be highly specific for their hosts, we note that SHFV-LVR and related viruses have been transmitted between primate species from presumptive African primate hosts into Asian macaques on several occasions [11]–[14], [31]. Recent work suggests that the capacity for SHFVs to infect multiple primate species is not unique to SHFV-LVR, as experimental infection of macaques with SHFV-krc1 resulted in viral replication and clinical disease (unpublished data). The biological properties of SHFV-krc1 and SHFV-krc2 in a natural host that we have identified herein may help explain the propensity of the SHFVs to infect primates of species other than their natural host. Future investigation of these viruses should provide further insight into the full extent of their cross-species transmission potential.

**Figure 8:**
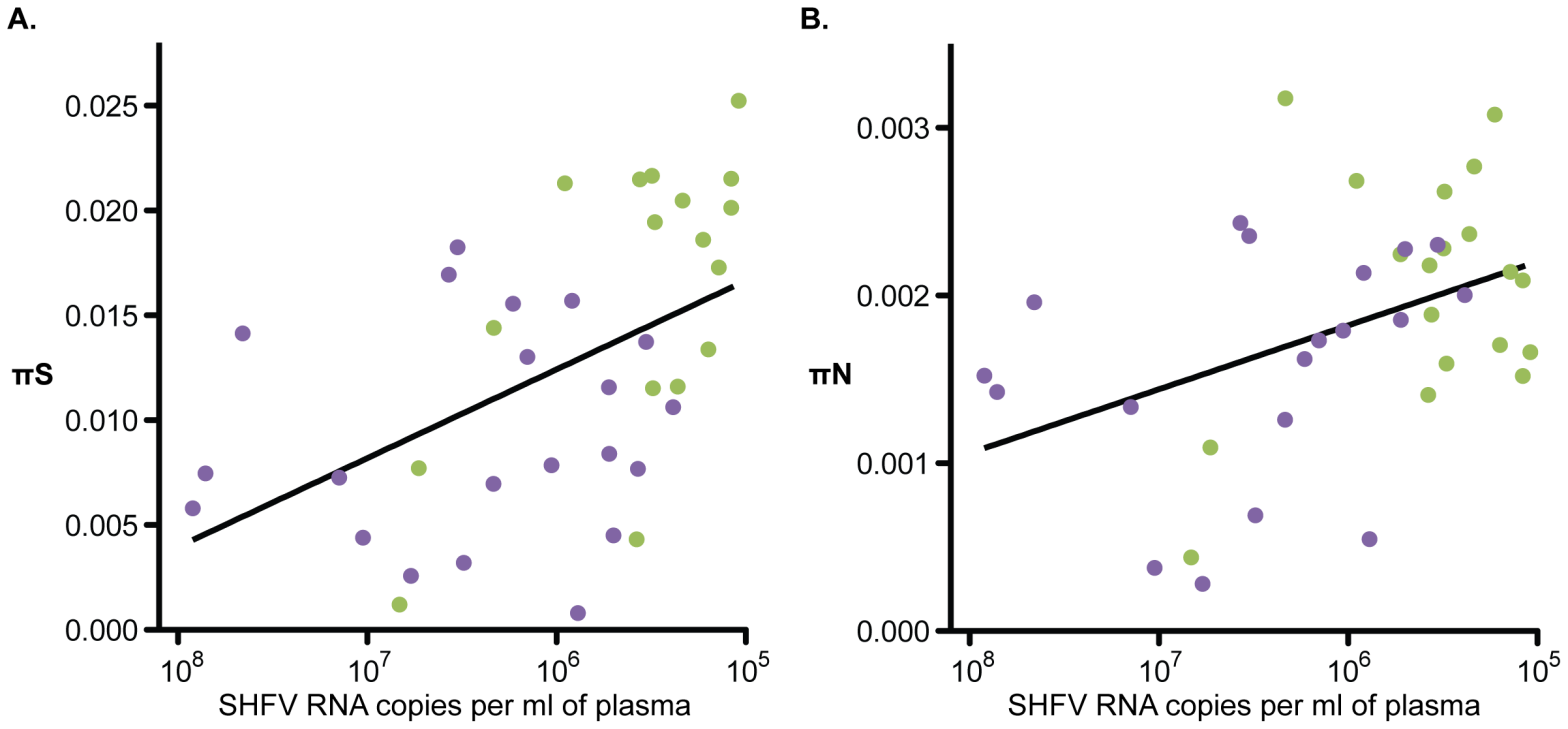
Relationship between viral load and nucleotide diversity. (A) Synonymous nucleotide diversity (πS) and (B) nonsynonymous nucleotide diversity (πN) were plotted against log-transformed viral loads for both SHFV-krc1 (green) and SHFV-krc2 (purple) infections. A significant correlation between nucleotide diversity and viral load was found for both πS (r2 = 0.2465, p = 0.0015) and πN (r2 = 0.1749, p = 0.0090).

Our analysis shows that SHFV-krc1 and SHFV-krc2 are not merely highly divergent forms of the same virus, but in fact possess unique and distinct biological properties. Nucleotide diversity was consistently higher in SHFV-krc1 than in SHFV-krc2. This is likely a result of the higher viral loads observed for SHFV-krc1, reflecting more extensive viral replication and a correspondingly higher rate of accumulation of within-host mutations [40]. This hypothesis is supported by positive correlations between viral load and both synonymous and non-synonymous nucleotide diversity (Figure 8). Interestingly, viral load and nucleotide diversity for both SHFV-krc1 and SHFV-krc2 were not significantly impacted by the presence of the other virus (Figure 3). When viewed in light of the competitive exclusion principle [41] this finding suggests that the two viruses may occupy discrete niches within the red colobus host (*e.g.* tissue tropisms), possibly resulting in distinct aspects of infection that could contribute to the observed differences in infection frequency (Figure 2) and viral burden (Figure 3).

The most significant difference in nucleotide diversity that we observed between SHFV-krc1 and SHFV-krc2 was found in ORF3 (Figures 6 and 7), which codes for the putative envelope glycoprotein GP3. GP3 of SHFV-krc1 and SHFV-krc2 appears similar in topology to GP3 of other arteriviruses, with predicted N- and C-terminal membrane-spanning domains separated by a heavily glycosylated ectodomain. While the precise function of GP3 in the arterivirus life-cycle remains elusive, GP3 is thought to be an important determinant of tissue tropism [42], [43]. GP3 is also immunogenic [44], [45] and glycans attached to the GP3 ectodomain may play a role in evasion of the humoral immune response through the shielding neutralizing antibody epitopes [46]. It is possible that GP3 has multiple functions, as GP3 of PRRSV and LDV have been found in both virion-associated and soluble secreted forms [47]–[51]. Our analysis revealed a distinct region of non-synonymous diversity suggestive of positive selection in ORF3 of SHFV-krc2 (codons 141–173) (Figure 7D). This region contained an unusually high density of acidic residues and multiple, variable putative *N*-glycosylation sites. Although a similar region was not found in ORF3 of SHFV-krc1, a unique peak of non-synonymous diversity was identified between codons 50–68 of ORF3 in SHFV-krc1 that was also suggestive of positive selection. Finally, another difference between SHFV-krc1 and SHFV-krc2 was that no signal sequence cleavage site could be identified in GP3 of any SHFV-krc1 variant, while a clear signal sequence cleavage site was found C-terminal to the first predicted transmembrane domain in GP3 of SHFV-krc2 [52]. The most likely explanation of this finding is that the signal sequence cleavage site of GP3 in SHFV-krc2 is not utilized, as has been shown for GP3 of EAV [44], [50].

Despite the differences we observed between SHFV-krc1 and SHFV-krc2 in ORF3, we found nearly identical patterns of non-synonymous and synonymous nucleotide diversity in ORF5, which – by analogy to other arteriviruses – codes for the major envelope glycoprotein GP5 [17], [53]. Two distinct peaks of non-synonymous diversity were found in the 5′-proximal region of ORF5, which corresponds to the protein's predicted ectodomain (Figure 7). This region of GP5 contains the primary neutralizing antibody epitope of PRRSV, EAV, and LDV [54]–[57], as well as an immunodominant “decoy” epitope in PRRSV that may serve to subvert neutralizing antibody responses [58]. These epitopes align closely with more 3′-proximal peak of non-synonymous diversity we identified in SHFV-krc1 and SHFV-krc2 (data not shown), suggesting that antibody pressure in the red colobus may select for escape mutations in SHFV-krc1 and SHFV-krc2, resulting in the observed genetic diversity of this region.

Glycans in this region of the GP5 ectodomain – in addition to aiding viral attachment through the binding of host molecules (*e.g.* sialoadhesin for PRRSV) [59] – are also implicated in evasion of humoral immune responses by arteriviruses. Pigs infected with PRRSV variants containing partially de-glycosylated GP5 mount significantly more robust neutralizing antibody responses [46], [60]. A similar observation was made for LDV in mice, and the abolishment of *N*-glycosylation sites in GP5 had the additional effect of altering the tissue tropism of these “neurotropic” LDV strains [61], [62]. Putative *N*-glycosylation sites were variably found in association with each peak of non-synonymous nucleotide diversity identified ORF5/GP5 of both SHFV-krc1 and SHFV-krc2 (Figure 7). However, in contrast to the GP5 ectodomains of PRRSV, EAV, and LDV, a highly conserved hydrophobic stretch of approximately thirty amino acids separated these two regions of diversity, and was predicted to form an additional transmembrane domain in both SHFV-krc1 and SHFV-krc2 [63]–[65]. A domain that spans the membrane once in this region would place the N-terminal portion of GP5 – including the region corresponding to the more 5′-proximal peak of non-synonymous nucleotide diversity – within the virion. While this possibility cannot be formally excluded, the high sequence diversity of this region – including multiple putative *N*-glycosylation sites – suggests that this scenario is unlikely. Nevertheless, it is conceivable that this region interacts extensively with the membrane of the virion and its functional significance, although obscure, is highlighted by its conservation across all other known simian arteriviruses including SHFV-LVR, SHFV-krtg1, and SHFV-krtg2 (data not shown).

The findings presented in this study show that SHFV variants contain high genetic diversity within their hosts. This presents the possibility that SHFV-krc1 or SHFV-krc2 could evolve rapidly within the red colobus, perhaps gaining virulence, similar to the recent emergence of highly pathogenic PRRSV in pigs in China and Southeast Asia [66], [67]. As the red colobus population of Kibale faces the stressors of deforestation and a changing climate, monitoring these infections may be important to the conservation of this already endangered wild primate [68].

Finally, these discoveries may facilitate research into many aspects of SHFV biology that remain poorly understood such as host range, tissue tropism, pathogenesis, immunity, and the question of persistence. Further characterization of these viruses in their natural free-living hosts (*eg.* serology) including long-term observation of this red colobus cohort in particular – and perhaps studies of SHFV in captive primates – should provide deeper insights into the biology of SHFVs and primate host-virus interactions in general.
