# Correction: High Genetic Diversity and Adaptive Potential of Two Simian Hemorrhagic Fever Viruses in a Wild Primate Population

**DOI:** 10.1371/journal.pone.0102939

**Published:** 2014-07-10

**Authors:** The PLOS ONE Staff

The following information is missing from the Funding section: This work was funded by NIH grant TW009237 as part of the joint NIH-NSF Ecology of Infectious Diseases program and the UK Economic and Social Research Council, and by the Wisconsin Partnership Program through the Wisconsin Center for Infectious Diseases, and the NIH (R01 AI077376-01). This publication was made possible in part by a grant (P51 RR000167) from the Office of Research Infrastructure Programs (ORIP), a component of the National Institutes of Health (NIH), to the Wisconsin National Primate Research Center (WNPRC), University of Wisconsin-Madison. This research was conducted in part at a facility constructed with support from Research Facilities Improvement Program grant numbers RR15459-01 and RR020141-01. ALB performed this work with support from the University of Wisconsin's Medical Scientist Training Program (MSTP) (grant T32GM008692). JHK performed this work as an employee of Tunnell Government Services, Inc. a subcontractor under Battelle's prime contract with NIAID, under Contract No. HHSN272200700016I. The authors thank the University of Wisconsin Department of Pathology and Laboratory Medicine and the WNPRC for funding and the use of its facilities and services. This publication's contents are solely the responsibility of the author(s) and do not necessarily represent the official views of ORIP, NIH, US Department of Health and Human Services, or of the institutions and companies affiliated with the authors. The funders of this research had no role in study design, data collection and analysis, decision to publish, or preparation of the manuscript.
